# Global health diplomacy at the intersection of trade and health in the COVID-19 era

**DOI:** 10.34172/hpp.2021.01

**Published:** 2021-02-07

**Authors:** Vijay Kumar Chattu, Shalini Pooransingh, Hamid Allahverdipour

**Affiliations:** ^1^Division of Occupational Medicine, Department of Medicine, Faculty of Medicine, University of Toronto, Toronto, ON, M5S 1A8, Canada; ^2^Institute of International Relations, The University of the West Indies, St. Augustine, Trinidad and Tobago; ^3^Department of Paraclinical Sciences, Faculty of Medical Sciences, The University of the West Indies, St. Augustine, Trinidad and Tobago; ^4^Department of Health Education and Promotion, Tabriz University of Medical Sciences, Tabriz, 14711, Iran

**Keywords:** Health, Diplomacy, Diet, Lifestyle, WHO, COVID-19, Health promotion, Non-communicable diseases

## Abstract

Global health diplomacy has gained significant importance and undoubtedly remained high on the agendas of many nations, regional and global platforms amid the coronavirus disease 2019 (COVID-19) pandemic. Many countries have realized the importance of the health sector and the value of a healthy workforce. However, there is little control on issues related to trade that impact on human health due to the dominance of profit-oriented business lobbies. A balance, however, needs to be struck between economic profits and a healthy global population. This paper aimed to highlight the importance of building capacity in global health diplomacy, especially during the COVID-19 pandemic so that health personnel may effectively negotiate on the multisectoral stage to secure the resources they need. The recent proposal to waive off certain provisions of the Trade-Related Aspects of Intellectual Property Rights (TRIPS) agreement for the prevention, containment and treatment of COVID-19 by India and South Africa at the World Trade Organization (WTO) presents an important opportunity for all governments to unite and stand up for public health, global solidarity, and equitable access at the international level so that both developed and developing nations may enjoy improved health outcomes related to the COVID-19 pandemic.

The importance of global health diplomacy (GHD) and particularly the prominence of the interdisciplinary concept involved in multisectoral and multi-level negotiations for global cooperation was highlighted by Ariansen et al.^1^ Their paper has highlighted “The Lancet’s year for Nutrition-2019” which included the EAT-Lancet Commissionand the Global Syndemic Commission^2^ emphasizing a revamp of global trade systems to ensure healthy and sustainable diets.

According to the 2017 Global Burden of Disease study, 11 million deaths and 255 million disability-adjusted life years were attributable to dietary risk factors highlighting the need for improving diet across nations.^3^ The coronavirus disease 2019 (COVID-19) pandemic has in fact sensitized many heads of states, ministers of non-health portfolios and the general public about the importance of health and healthy lifestyles. Though the epidemic of non-communicable diseases (NCDs) is not as formidable as the threat of infectious diseases, NCDs are silent killers contributing to premature deaths globally. As Ottersen et al. highlighted, the health community has failed to place the issue of health on the agenda of the World Trade Organization (WTO).^4^ The rapid rise in lifestyle-related diseases in middle-income countries where three-quarters of the world’s population resides suggests that the health of billions will benefit from tighter trade regulations. However, the trade agreements still facilitate unhealthy diets and lifestyles by prioritizing the profits over ill-health.^5^

Following the criticism of the trade agreements of the WTO which facilitates unhealthy lifestyles and unhealthy dietsby the Lancet Trade and health series, there was a welcome positive step on November 21, 2011 where Mr. Pascal Lamy, the Director of WTO and Dr. Haik Nikogosian, Head of the WHO Framework Convention of Tobacco Control (FCTC) met to review areas of intersection between trade and tobacco control which relied on cooperation between WTO and WHO.^6^

Indeed, the Oslo Ministerial Declaration was developed by foreign ministers without involvement from health ministers to raise the political profile of health.^7^ The health ministry itself is one of the poorly funded ministries in many governments, and the health ministers have no room for increasing the budget as they rely on their respective ministries of finance. The question arises as to how a health minister can compete for more funds against the profit-making ministries and industry sectors with strong food industry lobbies^2^ (e.g. beef, dairy, sugar, and ultra-processed food and beverages).

As a result of the COVID-19 pandemic, health has become a top priority on the agendas of national governments, regional and global platforms. Health ministers should be proactive and take the lead for negotiations that will ensure “Health in All Policies” (HiAP) and should demonstrate how with proper funding, a healthy society can actually contribute to the nation’s productivity. Addressing the underlying socio-economic determinants, gender and equity issues is possible only through dedicated funding and multi-sectoral collaboration. The role of GHD is paramount at the time when the global challenges such as climate change, poverty and food insecurity are major concerns for many developing nations in the global south. There is a need for collective action and collaboration among physicians, lawyers, policy makers, economists and professionals from other domains to put health on the agenda of non-medical policymaking global institutions such as WTO, other regional and UN bodies. The recent actions by the pharmaceutical corporations fighting for their exclusive rights on their patents with limited voluntary actions can potentially deteriorate the current pandemic situation. For example, it is worth noting that Thailand has sought to develop the capacity to engage in GHD, and although its active development is perhaps an exception. The Thailand case study offers three important lessons for other countries: (1) Developing capacity for both health and non-health actors so that both understand the health impact of non-health polices and vice versa which may lead to better understanding of trade negotiations; (2) Improving the interaction structure between related agencies can facilitate benefit from institutionalized and ad hoc agencies and (3) Finally an informal network is important in building the trust on which collaboration is centered. The capacity developed in Thailand aided the member states of the South-East Asia Region in developing and strengthening the capacity of their health and related professionals.^8^

The Ottawa Charter for Health Promotion in 1986 emphasized that health promotion is not just the responsibility of the health sector but goes beyond healthy lifestyles to well-being as a collective action. The requirements of health improvement require certain basic pre-requisites such as peace, shelter, education, food, income, a stable-ecosystem, sustainable resources, social justice and equity which cannot be achieved by the health sector alone. Political, economic, social, cultural, environmental, behavioural and biological factors can all favour health or adversely affect it. The Charter further advocates that health promotion demands coordinated action by all concerned such as governments, health and other social and economic sectors, by non-governmental and voluntary organizations, local authorities, industry and the media.^9^ This Charter led the foundation for the UN- MDGs and the subsequent Sustainable Development Goals, which advocates for global partnerships, ending poverty, health-related goals, climate change and security.^10^ As highlighted by Dugani et al, a concerted approach is required to reduce the cost of medicines and increase access to affordable health care.^11^ Therefore, to address these inter-related and multisectoral challenges, especially to negotiate the issues between trade and health, we need the successful practice of GHD specifically to help the low- and middle-income countries as they rely on developed nations for guidance and financial assistance.

Without strong political will and accountability, GHD has limited success in reversing the NCD epidemic. The success of global Framework Convention on Tobacco Control (FCTC) is an excellent example of GHD, and another good example is the Port of Spain Declaration from the CARICOM countries with a high-level commitment from the heads of state.^12^ The role of civil society organization (CSO) and their active participation such as Healthy Caribbean Coalition^13^ supported by NCD Alliance advocated the importance of strategic engagement with non-health ministries, such as the Ministry of Finance, to encourage actions that will lead to ‘HiAP.’ As a result, Barbados became the first country in the Caribbean to demonstrate its commitment by supporting the WHO’s Best Buys^14^ by reducing sugar consumption through effective taxation on sugar-sweetened beverages as part of a broader NCDs prevention strategy. This strategy was implemented by other countries - Chile, Finland, France, French Polynesia, Hungary, Mauritius, Mexico, Samoa, and Tonga.^15^ Similarly, Sweden, Germany, Qatar, and Brazil have developed dietary guidelines that promote environmentally sustainable diets and eating patterns that ensure food security, improve diet quality, human health and wellbeing, social equity, and respond to climate change challenges.^2^

Up until 2015, a total of 75 countries have developed a national salt reduction strategy to reduce salt intake by 30% by 2025^16^ as part of WHO’s Best Buys. All these developments on taxation of sugar, salt, tobacco, alcohol and others are the result of an ongoing GHD between multiple stakeholders (WHO, WTO, CSOs, member countries etc). However, the success was possible only because the nations chose to prioritize health over profits. Unfortunately, this solution may not be applicable to low- and middle-income developing countries with a triple burden of infectious diseases, NCDs and nutrition-related conditions compounded by poverty, inequities and the impacts of climate change.

Countries need to persist and come up with bold, innovative proposals to benefit their populations.

India and South Africa have developed a proposal to the WTO to waive some of the requirements under the Agreement on Trade-Related Aspects of Intellectual Property Rights (TRIPS) to support the manufacture of medical products to aid the COVID-19 pandemic response.^17^ This proposal if granted would allow the WTO members to choose to neither grant nor enforce patents and other intellectual property (IP) related to COVID-19 drugs, vaccines, diagnostics, and other technologies including masks and ventilation during this phase of the pandemic. However, during the WTO TRIPS Council meeting on October 16, 2020 nine WTO members including the European Union, Australia, Brazil, Canada, Japan, Norway, Switzerland, United Kingdom and United States did not support the proposal though 100 countries showed support.^18^ It therefore appears that there is hesitation for international cooperation to address the crisis and to promote equitable access across all nations.^19^ There is a call for the WTO to suspend patents related to COVID-19 vaccine development. The pharmaceutical industry is engaging with the WHO COVID-19 Technology Access Pool (C-TAP) which aims to encourage the voluntary contribution of IP, technology and scale up manufacturing and supply of COVID-19 health technologies.

An example of overcoming IP monopolies by pharmaceutical industry is the case of antiretrovirals for HIV. To oppose the monopolies of the pharmaceutical industry, there were many ‘access-to-medicines’ movements led by patient activists, civil society and health-rights groups who stood up to governments’ inaction to secure HIV medicines; they finally succeeded in getting relief from patents. This has resulted in a significant decrease in the prices of HIV medicines (over $10000 /person/year) which dropped by 99% over a decade by allowing the production of generic drugs by developing countries.^18^ Today a similar scenario presents itself with COVID-19; if the TRIPS waiver proposal is approved, the access to essential COVID-19 medicines, technologies and diagnostics should improve drastically. Therefore, to counterbalance the immense power of the multi-national companies/ corporations with their vested interests and profit-seeking agenda, there is a great need for involving physicians trained in GHD in shaping the global trade policies as well as being the uncontested authorities in the domain of health.

## Conclusion

GHD has undoubtedly come to the fore, and apart from highlighting Global Health, it has derived attention from fields such as international relations, foreign policy, international law, and economics. The concept is growing day by day, and the need for professionalization of GHD is pressing. Special programs/courses in GHD are offered at individual premier institutions such as Graduate Institute Geneva, Diplo Foundation at Malta, National School of Public Health Fiocruz in Brazil, State University of New York, University of Oxford in the UK, Nova University of Lisbon in Portugal, WHO-EMRO in Egypt and the University of Toronto in Canada to name some. However, there is a need for cross-disciplinary programs in GHD. GHD should be an integrated part of medical professional studies and other health-related areas such as global health, health policy, health economics, health leadership, health administration, etc. GHD as an interdisciplinary profession can equip the medical/ health professionals with the skills necessary to engage in negotiating trade policies which can impact the health of billions. Governments need to fulfil the core obligations of its citizens by protecting public health, implement appropriate policies for healthy life-styles (through taxation on unhealthy foods, beverages etc) and ensuring access to medicines for all. Particularly key to this argument is the current issue of ensuring access to the COVID-19 vaccine to all. It is easy to talk of inequalities and inequities and include them in policies. However here is an opportunity for the world to show its solidarity for ‘Health For All’ and nations should strive to find solutions to ensure equitable access to the vaccine while allowing for profit making by the industry and this is where GHD has a major role to positively impact health outcomes during the COVID-19 pandemic.

## Acknowledgements

The authors thank the reviewers for their critical feedback to improve the quality of this manuscript.

## Funding

Nil.

## Competing interests

The authors declare that there are no conflicts of interests.

## Ethical Approval

Not applicable.

## Authors’ contributions

VKC involved in concept, design, literature search. VKC and SP collected the required information, did initial manuscript preparation and manuscript editing. HA edited the manuscript, reviewed and provided critical comments for the final version. All the three authors VKC, SP and HA have approved the final version.
